# Serum Level of Matrix Metalloproteinase-3 in Patients with Oral Lichen Planus

**Published:** 2012-01-01

**Authors:** M Farzin, M Mardani, J Ghabanchi, M J Fattahi, M Rezaee, S T Heydari, A Andisheh Tadbir

**Affiliations:** 1 Department of Prosthodontics, Dental School, Shiraz University of Medical Sciences, Shiraz, Iran; 2 Department of Oral Medicine, Dental School, Shiraz University of Medical Sciences, Shiraz, Iran; 3 Shiraz Institute for Cancer Research Shiraz University of Medical Sciences, Shiraz, Iran; 4 Health Policy Research Center, Shiraz University of Medical Sciences, Shiraz, Iran; 5 Department of Oral Pathology, Dental School, Shiraz University of Medical Sciences, Shiraz, Iran

**Keywords:** Oral, Lichen planus, Matrix metalloproteinase-3

## Abstract

**Background:**

Oral Lichen planus (OLP) is a chronic lesion of the oral mucosa with unknown origin. Basement membrane changes are common in OLP and may be mediated by proteases such as matrix metalloproteinase (MMPs) and mast cell chymase. The aim of our study was to evaluate the level of serum MMP-3 in OLP com­pared to normal individuals and assess its clinical significance.

**Methods:**

Thirty four serum samples from patients diagnosed with OLP (12 males, 22 females, age: 42.2±10.8 years) and 34 serum samples from healthy control subjects (11 males, 23 females, age: 42.5±13.3 years) were collected and MMP-3 concentration was measured by ELISA.

**Results:**

The serum MMP-3 level in OLP patients was higher (21.64±24.31 ng/ml) compared with healthy con­trols (16.52±23.63 ng/ml), but showed no statistically significant difference. A statistically significant difference was demonstrated between the two types of OLP, being more pronounced in the erosive/atrophic form 6).

**Conclusion:**

The different clinical appearances of OLP are associated with significant differences in MMP-3 serum level.

## Introduction

Oral Lichen planus (OLP) is a chronic lesion of the oral mucosa, with a prevalence of 0.1% to 4%.[1] Clinically, OLP has a distinct morphology with two typical forms: atrophic-erosive lesions with or without concomitant reticular lesions, and reticular and/or plaque lesions.[2]

The cause of Lichen planus is unknown, but it is likely that both endogenous genetic and exogenous environmental components such as drugs or infec­tions may interact to elicit the disease. Cell-mediated immunity plays the major role in triggering the clini­cal expression of the disease.[3][4][5]

Basement membrane (BM) changes are common in OLP and comprise breaks, branches and duplica­tions. BM damage in OLP may be mediated by prote­ases such as matrix metalloproteinase (MMPs) and mast cell chymase.[6]

MMPs are a large family of zinc-dependent endopeptidases, which are capable of digesting ex­tracellular matrix and BM component.[7] MMPs gener­ally consist of a prodomain, a catalytic domain, a hinge region, and a hemopexin domain. They are ei­ther secreted from the cell or anchored to the plasma membrane. On the basis of substrate specificity, se­quence similarity, and domain organization, verte­brate MMPs are divided into six groups: collagenases, gelatinases, stromelysins, matrilysins, membrane-type MMPs and others.[8]

MMP-3 is a stromelysin which degrades many noncollagenous matrix components such as proteoglycans, fibronectin, laminin and gelatin in addition to type III , IV and V collagen and also activates interstitial procol-lagenase (proMMP-1) and progelatinase B (proMMP- 9).[9] A number of metalloproteinases have been identi­fied in blood serum that are categorized mainly in four groups: a) collagenases (MMP-1, MMP-8, MMP-13, MMP-18), b) gelatinases (MMP-2, MMP-9), c) stromelysins (MMP-3, MMP-10) and d) membranebound metalloproteinases (MMP-14, MMP-15, MMP-16, MMP-17, MMP-24, MMP-25).[10]

Previous studies using immunohistochemistry showed that MMP-2 and MMP-3 were mainly found in OLP epithelium, but no one has elucidated the cir­culating serum level of MMP-3 in OLP. Therefore, the aim of our study was to evaluate the level of se­rum MMP-3 in OLP compared to normal individuals and assess its clinical significance.

## Materials and Methods

For the purpose of this study, 34 serum samples from patients diagnosed with OLP (12 males, 22 females, age: 42.2±10.8 years) and 34 serum samples from healthy control subjects (11 males, 23 females, age: 42.5±13.3 years) were collected. All the study patients were admitted to Oral Medicine Department of Shiraz University of Medical Sciences and OLP was diagnosed clinically and histopathologically in all of these patients.

Control cases were healthy blood donors, who were matched for age and sex. Exclusion criteria in­cluded the presence of any systemic disease, use of corticosteroid or non-steroid anti-inflammatory medi­cation, or a history of chronic pulmonary disease or malignancy of any type. All participants were in­formed about the research study and agreed to par­ticipate by signing an informed consent form. The types of OLP were subclassified into two clinical forms: reticular and/or plaque lesions (25 cases) and erosive/atrophic lesions (9 cases).

Serum samples were obtained from clotted blood following centrifugation at 4°C and stored at -80°C until analysis. MMP-3 concentrations were measured by ELISA in accordance with the manufacturer's in­structions (BMS2014/2 BenderMed Systems GmbH, Germany). Statistical analysis was performed by us­ing Mann-Whitney and Chi-Square tests. A p value less than 0.05 was considered significant.

## Results

Histologic examination of all OLP specimens showed typical findings of this disease, including a band-like, mainly lymphocytic infiltrate in the connective tissue adjacent to the epithelial basement membrane, lique­faction degeneration of the basement membrane, and destruction of basal keratinocytes. There were 22 (64.7%) females and 12 (35.3%) males diagnosed with OLP in our study.

The serum MMP-3 level in OLP patients was higher (21.6±24.3 ng/ml) compared with healthy con­trols (13.5±17.9 ng/ml), but showed no statistically significant difference (p=0.227).

After adjustment for sex, a statistically significant difference was demonstrated between the two types of OLP, being more pronounced in the erosive/atrophic form, correlating with the severity of this malady (Table 1 ,p<0.001). The serum level of MMP-3 was statistically higher in male patients (28.0±23.2 ng/ml) than in females (12.2±18.8 ng/ml, p<0.001).

**Table 1:** Serum level of MMP-3 in different groups

**Group **	**Number **	**Level of MMP-3 (ng/ml)^a^**
Control	34	13.5±17.9
Reticular OLP	25	9.9±12.3
Erosive OLP	9	49.8±23.2

^a^ The difference of mean MMP-3 between control and reticular OLP were not significance but mean MMP-3 in erosive OLP dramatically was higher than other groups (p<0.001).

## Discussion

The pathogenesis of OLP features a complex series of interactions between inflammatory cells, chemokines and cytokines that ultimately determine the apoptosis of basal keratinocytes, triggered by the contacts be­tween CD8+- activated lymphocytes and an unknown antigen expressed on the surface of basal cells.[11][12]BM degradation, which allows lymphocytes to mi­grate, involves proteolytic enzymes called MMPs.[6][13]MMPs involved in cell migration, angiogenesis and proteolytic activation of growth factors, events needed in normal tissue remodeling as well as wound healing and tumor invasion.[14] The first study investi­gating the relation of LP and MMPs was by Giannelli et al.,[15] They reported increased MMP-2 expression in acute stages of LP and suggested that an altered balance between MMP-2 and TIMP-2 may play a role in the destruction of BM. In 1998, the results of a study comparing OLP, dysplasia and squamous cell carcinoma (SCC) revealed that MMP-1, MMP-2, and MMP-3 expression were lower in OLP than SCC, and MMPs and TIMPs were clearly upregulated during invasion in oral SCC.[16]

Later, Zhou et al. reported MMP-2 and MMP-3 expression in OLP epithelium and increased MMP-9 expression in the inflammatory infiltrate cells. They suggested that MMPs may act synergistically to de­grade the epithelial BM in OLP.[5] Kim et al. reported that under upregulation by bone morphogenetic pro­tein BMP-4, both MMP-1 and MMP-3 expression in OLP may induce epithelial cells acantholysis and lead to erosive changes.[17]

Mazzarella et al. showed that the overall levels of expression of MMP mRNAs were higher in erosive OLP than in the reticular form. Moreover, MMP-1 and MMP-3 may be principally associated with ero­sion development.[18] Tsai et al. were the first who elu­cidated the circulating plasma expression of MMPs in OLP.[19] They reported that MMP-2 overexpression in OLP is consistent with its upregulation in peripheral serum. In the present study we showed that serum level of MMP3 was not significantly higher than healthy controls.

Furthermore, serum MMP-3 levels tended to change with the switching of the clinical subtypes of OLP, particularly increasing in the erosive/atrophic of the disease. MMP families can do the breakdown of intercellular bridge and acantolysis.[17] Our results suggested that MMP-3 seems to play a key role in the transformation of reticular to erosive form, possibly by inducing acantholysis and the low level of MMP3 in reticular OLP could therefore explain the absence of erosions in this clinical form.

Our findings was in accordance with Mazzarella et al. which reported that expression of MMP-3 mRNAs were higher in erosive Lichen planus than in the reticular form. A permanent or prolonged presence of high MMP-3 amounts in serum may contribute to malignant transformation of OLP lesions, It is known that MMP-3 has a role in oncogenesis and expressed in OSCC,[20][21] as well as that the erosive/atrophic form of OLP has a greater rate of malignant evolution in comparison with the reticular form.[22]

In our study there were statistically significant dif­ferences between male and female patients in terms of the serum MMP-3 levels. Probably, this difference was due to the fact that erosive form was more com­mon in males than females in this study. In conclu­sion we showed that the different clinical appearances of OLP were associated with significant differences in MMP-3 serum level.
